# Evaluation of Thermal-Mechanical Properties of Bio-Oil Regenerated Aged Asphalt

**DOI:** 10.3390/ma11112224

**Published:** 2018-11-08

**Authors:** Tianyuan Yang, Meizhu Chen, Xinxing Zhou, Jun Xie

**Affiliations:** 1 State Key Laboratory of Silicate Materials for Architectures, Wuhan University of Technology, Wuhan 430070, China; chenmzh@whut.edu.cn; 2 Key Laboratory of Highway Construction and Maintenance Technology in Loess Region of the Ministry of Transport, Shanxi Transportation Research Institute, Taiyuan 030006, China; zxx09432338@whut.edu.cn

**Keywords:** thermal–mechanical properties, bio-oil, regeneration, aged asphalt, molecular dynamic simulation

## Abstract

Different proportions of bio-oil (5, 10, 15, and 20 wt%) were added into aged asphalt for its regeneration. Molecular dynamic simulations were used to measure the thermal and mechanical performances of bio-oil regenerated aged asphalt (BRAA). A new, simplified BRAA model was built to calculate the specific heat capacity, thermal expansion coefficient, elastic constant, shear modulus, bulk modulus, and Young’s modulus. Simulation results showed that the thermal expansion coefficient (CTE α) of asphalt at 298 K decreased by 10% after aging. Bio-oil of 5 wt% could make the CTE α restore to the original level of base asphalt, while the addition of bio-oil would further decrease the specific heat capacity of aged asphalt. The shear modulus (G), Young’s modulus (K) and bulk modulus (E) of asphalt increased after aging and decreased with the increasing amount of bio-oil. According to the calculated E/G value, the ductility of aged asphalt increased by 6.0% with the addition of 10 wt% bio-oil, while over 15 wt% bio-oil would make the ductility of BRAA decrease. In summary, the regeneration effects of bio-oil to the thermal expansion coefficient, flexibility, and ductility of aged asphalt had been proven, while excessive bio-oil would decrease the thermal stability of asphalt.

## 1. Introduction

Asphalt mixture is a common pavement material. It provides a comfortable driving environment for vehicles. However, the pavement performance of asphalt mixture constantly decreases during service process. One of the key reasons is that asphalt, as a binder, is gradually aging under the influence of oxygen and ultraviolet [1,2,3]. In order to recycle and reuse the aged asphalt, a considerable amount of research has been done on the regeneration of aged asphalt. In 1915, asphalt recycling technology was first explored by American Warren Bother who reused the aged asphalt by heating waste asphalt mixture [4]. After 1956, due to the initial formation of the US domestic high-grade road network, the price of petroleum crude oil rose rapidly [5]. During the period from 1970 to 1989, the global oil crisis occurred and crude oil price continued to rise. The cost of new asphalt pavement increased greatly, thus, people paid more attention to asphalt recycling technology. Regarding the recycling mechanism of aged asphalt, the regeneration of aged asphalt can be considered as the reverse process of asphalt aging. By means of adding specific substances, the composition of asphalt is adjusted to reverse its performance and return to an appropriate state that can be reused [6,7,8]. Not only rheological properties but also changes in structure were considered as the indicators for the evaluation of asphalt regeneration effect. More testing methods, such as NMR, were used to investigate the additive’s effects on aged asphalt [9].

Bio-oil is extracted from waste wood under 500 °C through thermal pyrolysis [10,11,12]. It has been a recently considered modifier applied to asphalt [13,14]. The main ingredient of bio-oil is close to petroleum asphalt, which makes bio-oil compatible with asphalt. Extensive research has been done to investigate the properties of bio-oil modified asphalt [15,16,17]. Williams [18] studied the physical and chemical properties of bio-oil and the physic-chemical behavior during thermal cracking. Yang [19] prepared the bio-oil modified asphalt and evaluated its properties. Guarin [20] investigated the rheological and chemical characterization of bio-oil modified asphalt and compared effects of several bio-oil materials in modification. The indices of viscosity, rutting and fatigue factors of bio-asphalt were investigated [21] and the optimal bio-asphalt production process was eventually determined by orthogonal experimental method. It has been proven that bio-oil could improve the diffusion and rheological properties of asphalt. Although research has been done with respects to the rheological, chemical or constructional properties of bio-asphalt [22,23], there is little in the way of literature introducing the regeneration effect of bio-oil recycled aged asphalt.

In this research, bio-oil was added into aged asphalt. Thermal-mechanical properties were studied to evaluate its rejuvenating effects on aged asphalt by means of molecular dynamics (MD) simulation. MD is a computer simulation method used to study the physical motion of atoms and molecules. Atoms and molecules are allowed to interact for a fixed period of time, so that the dynamic evolution of the system can be seen. In the most common version, the trajectories of atoms and molecules are determined by numerically solving Newton’s equations of motion for a system of interacting particles, where the forces between the particles and their potential energies are usually calculated using the inter-atomic potential or the molecular mechanics field. This method was originally developed in the field of theoretical physics in the late 1950s [24,25], but is now mainly used in chemical physics, materials science, and bio-molecules modeling. Molecular models and simulation techniques can be used to study the interaction between asphaltenes, resins, saturates, and aromatics in asphalt, and to analyze the relationship between composition, structure, and performance [26,27,28]. Computers are used to calculate the model, predict the performance of the asphalt, and guide the design of the asphalt material. The molecular model of asphalt is divided into three categories: (1) One-component asphalt model; (2) three-component model (asphaltenes, soft asphalt, and resins) [29,30,31]; (3) four-component model (asphaltenes, aromatics, resins, and saturates) [32]. The physical properties, rheological properties, thermodynamic properties, and dissolution properties of asphalt can be predicted, in order to guide asphalt production.

In this research, molecular models of base asphalt, aged asphalt, and bio-oil regenerated aged asphalt (BRAA) were constructed. These models were simulated in a range of temperature. After that, the thermal–mechanical properties, including thermal expansion coefficient, heat capacity, elastic constant, shear modulus, Young’s modulus, and bulk modulus were calculated. Among these properties, the thermal expansion coefficient and heat capacity reflect the performance of the asphalt during heating, which both determine the applicable temperature of the asphalt. The moduli affect the mechanical performances of asphalt, related to its resistance to damage.

## 2. Molecular Models and Methods

### 2.1. Modeling and Simulations Details

**Figure 1:**
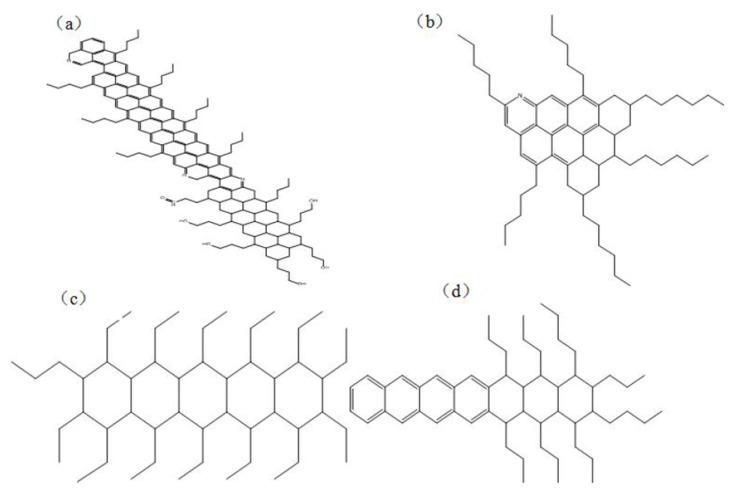
Components of asphalt: (**a**) asphaltenes; (**b**) resins; (**c**) saturates; (**d**) aromatics.

The components of asphalt include four main classes of fractions: Asphaltenes, resins, saturates and, aromatics. Asphaltenes consist of high molecular weight phenols and heterocyclic compounds. Resins consist of high molecular weight phenols and carboxylic acids produced by partial oxidation of the material. Saturates correlate with softening point of asphalt. Aromatics consist of partially hydrogenated polycyclic aromatic compounds. In Figure 1, the molecules were selected to represent asphaltenes, resins, saturates, and aromatics to construct the asphalt model. Moreover, the aged asphalt was selected by aging 50 h [33,34].

The main components of bio-oil extracted from waste wood were acetic acid, 1-carboxy-2-propanone, and methanol [35]. Bio-oil was mixed with aged asphalt to prepare BRAA by the mass fraction of 5%, 10%, 15%, and 20% to asphalt, respectively. Finally, six asphalt systems were obtained. Their compositions are shown in Table 1.

**Figure 2:**
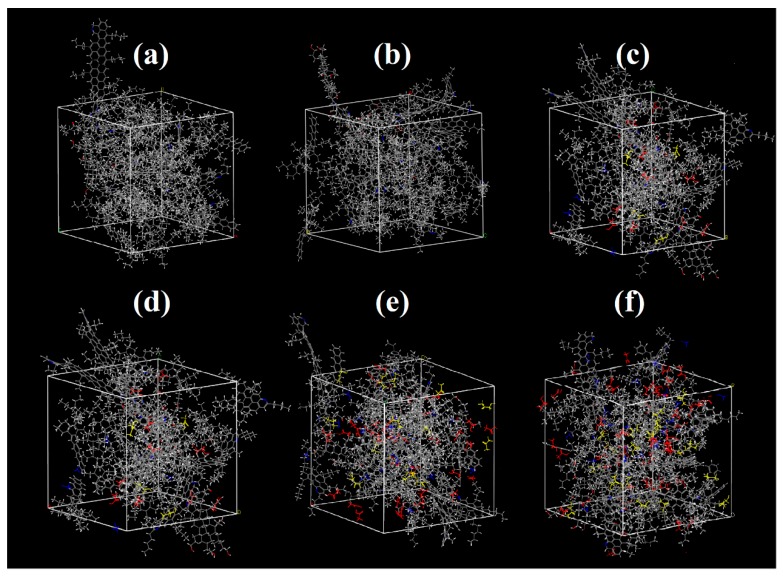
Models of asphalt systems: (**a**) asphalt; (**b**) aged asphalt; (**c**) 5% BRAA; (**d**) 10% BRAA; (**e**) 15% BRAA; (**f**) 20% BRAA.

The simulations were performed using Materials Studio, the software for simulating and modeling materials. Firstly, 3D models of the molecules showed above were constructed. Then, amorphous cell module was used to mix the components and to build the asphalt systems. The initial densities were set to be 1.0 g/cm^3^. Pressure was set at 101.325 kPa (1.0 atm). The asphalt systems were first relaxed to induce the system energy using the geometry optimization tool of Forcite module, then relaxed to state equilibrium using the dynamic tool with the parameters set to be isobaric-isothermal ensemble (NPT), 1.0 fs time step and 200 ps simulation time. After that, the systems were relaxed to state equilibrium with canonical ensemble (NVT), 1.0 fs time step and 200 ps simulation time. Condensed-phase optimized molecular potentials for atomistic simulation studies (COMPASS) force filed were chosen in all molecular simulations and minimization processes in this research. The following processes were performed to simulate the physical state of asphalt systems and to calculate the thermal-mechanical properties. Simulation temperature ranged from 223 to 448 K. The six asphalt systems are shown in Figure 2.

### 2.2. Methods (Calculation Details)

#### 2.2.1. Specific Heat Capacity

Specific heat capacity (*C_p_*) is a measurable physical quantity equal to the ratio of the heat added to (or removed from) an object to the resulting temperature change [36]. During the heating process, the higher specific heat capacity slowed the asphalt temperature rise more slowly, which means that more energy will be consumed during asphalt heating. Otherwise, asphalt with higher specific heat capacity will be less susceptible to ambient temperature.

In this research, asphalt systems were relaxed to state equilibrium at the same pressure (1.0 atm) and different temperatures (223 to 448 K). The systems would have different properties at different temperatures. The specific heat capacity of asphalt could be analyzed by comparing the enthalpies of asphalt systems at different temperatures, as follows:

(1) $$C_{p}=\frac{H}{T_{p}}=\frac{\left(E+PV\right)}{T_{p}}=\frac{1}{k_{B}T^{2}}\left(\langle {\left(E+PV\right)}^{2}\rangle -{\langle E+PV\rangle }^{2}\right)$$

where *C_p_* is specific heat; *E* is energy; *P*, *V*, and *T* are pressure, volume, and temperature, respectively; and $k_{B}$ is Boltzmann content. The *E*, *P*, *V*, and *T* could be obtained from result texts after dynamic simulations.

#### 2.2.2. Thermal Expansion Coefficient

Thermal expansion is the tendency of matter to change in shape, area, and volume in response to a change in temperature [37]. In this research, asphalt systems were relaxed to state equilibrium at different temperature (223 to 448 K) to simulate the heating process of asphalt. With the change in temperature, the volumes of asphalt systems simultaneously changed. The volumetric thermal expansion coefficient (CTE α) could be calculated by the following equation:

(2) $$\alpha =\frac{1}{V}{\left(\frac{\partial V}{\partial T}\right)}_{P}$$

where *V*, *T*, and *P* are volume, temperature, and pressure, respectively. The *V*, *T*, and *P* could be obtained from result texts after dynamic simulations.

#### 2.2.3. Elastic Constant

After the asphalt systems relaxed to state equilibrium, the elastic constants of systems were calculated by Mechanical Property tool of Forcite module. A GPa of 0.03 was chosen to be the maximum strain applying to the asphalt systems in this simulation. For elastic materials, the elasticity could be evaluated by Hook’s law:

(3) $$\sigma_{i}=C_{ij}\varepsilon_{j}$$

where *i*, *j* = 1, 2, 3. $\sigma_{i}$ is the stress vector, while $\varepsilon_{j}$ is the strain vector; C*_ij_* is the six-dimensional stiffness matrix. The stress components could be calculated as follows:

(4) $$\sigma_{ij}=-\frac{1}{V}\underset{k}{\sum }\left[m^{k}\left(u_{i}^{k}u_{j}^{k}\right)+\frac{1}{2}\underset{l\neq k}{\sum }\left(r_{i}^{kl}\right)f_{j}^{lk}\right]$$

where *V* is the volume; *m^k^* and *u^k^* are the mass and velocity of the *k*th particle, respectively; *r* is the distance between the *k*th and the first particles; *f* is the force exerted on the first particle by the *k*th particle. Lame coefficient *λ* and *μ* can be calculated by the following:

(5) $$\lambda =\frac{1}{6}(C_{12}+C_{13}+C_{21}+C_{23}+C_{31}+C_{32})\approx \frac{1}{3}\left(C_{12}+C_{23}+C_{13}\right)$$

(6) $$\mu =\frac{1}{3}\left(C_{44}+C_{55}+C_{66}\right)$$

(7) $$\lambda +2\mu =\frac{1}{3}\left(C_{11}+C_{22}+C_{33}\right)$$

#### 2.2.4. Young’s Modulus, Shear Modulus, and Bulk Modulus

Young’s modulus, denoted by *K*, is a mechanical property of linear elastic solid materials, measuring the stiffness of a solid material. It defines the relationship between stress and strain in the material, which will be more or less dependent on temperature. Shear modulus, always denoted by *G*, is defined as the ratio of shear stress to the shear strain [38]. Bulk modulus, denoted by *E*, is defined as the ratio of the infinitesimal pressure increase to the resulting relative decrease of the volume. They could be calculated as follows:

(8) $$K=\frac{\mu \left(3\lambda +2\mu \right)}{\lambda +\mu }$$

(9) G=μ

(10) $$E=\lambda +\frac{2}{3}\mu$$

where *K*, *G*, and *E* are Young’s modulus, shear modulus, and bulk modulus, respectively; λ and μ are Lame coefficients.

## 3. Results and Discussions

### 3.1. Thermal Properties of BRAA

**Figure 3:**
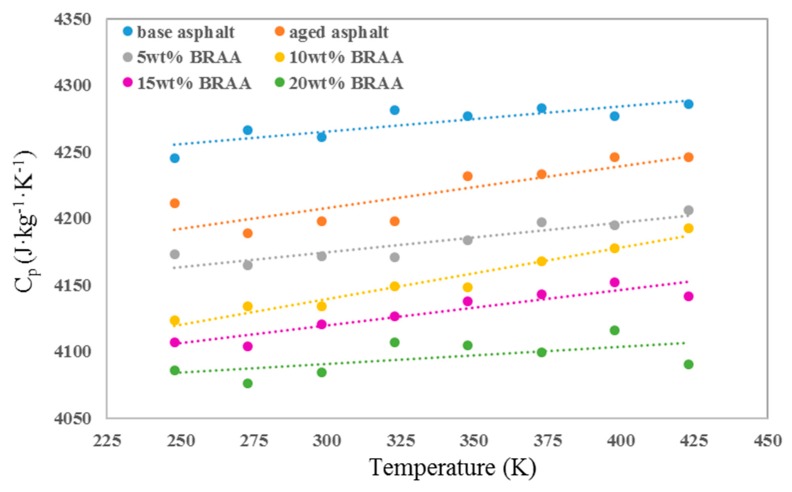
Specific heat capacities of asphalt systems.

The parameters of asphalt, aged asphalt, and BRAA were calculated by molecular dynamic simulations at different temperatures. As shown in Figure 3, the specific heat capacity (*C_p_*) of asphalt systems showed an upward trend with temperature rises. Moreover, it decreased with aging condition and the addition of the bio-oil. The results indicated that the bio-oil renders asphalt more sensitive to temperature. Furthermore, aging would weaken the temperature sensitive of asphalt binder, and the relationship between *C_p_* and temperature is linear.

**Figure 4:**
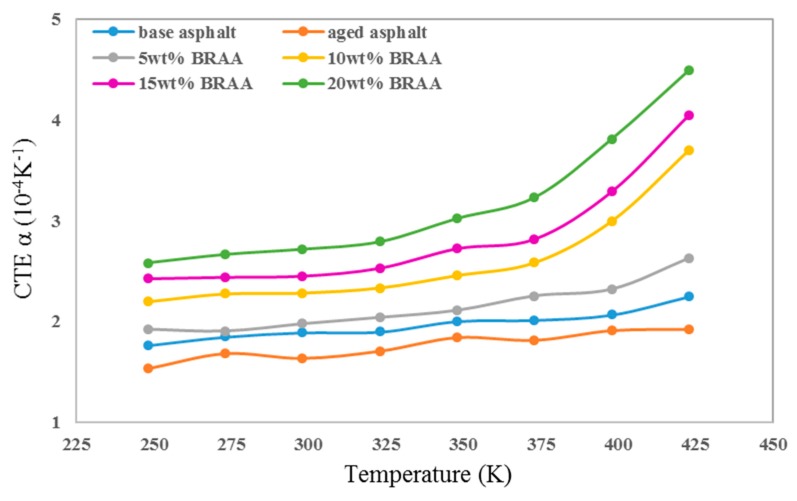
Thermal expansion coefficients of asphalt systems.

Figure 4 showed the thermal expansion coefficient (CTE α) of asphalt, aged asphalt, and BRAA at different temperatures. As can be seen, the aged asphalt had the lowest CTE α, while the 20 wt% BRAA showed the highest. The CTE α of asphalt at 298 K decreased by 10.0% after aging. The addition of 5 wt% bio-oil could increase the CTE α of aged asphalt by 13.4% and make it even higher than that of base asphalt. Excessive bio-oil would further increase the CTE α of asphalt. However, when the mass fraction of bio-oil is more than 10%, the CTE α of BRAA would significantly increase over 350 K. The results indicated that the rejuvenating effect of bio-oil on aged asphalt shows an optimum dosage of 10% regarding the CTE α. Excessive bio-oil would decrease the thermal stability of asphalt and induce thermal expansion.

As conclusions, the CTE α of aged asphalt could be improved and the *C_p_* would be decreased with the addition of bio-oil. Bio-oil of 5 wt% would be the suitable choice to regenerate the aged asphalt. CTE α of aged asphalt could restore the original level with the addition of 5 wt% bio-oil, while the thermal stability of asphalt would decrease when the amount of bio-oil was over 10 wt%.

### 3.2. Mechanical Properties of BRAA

**Figure 5:**
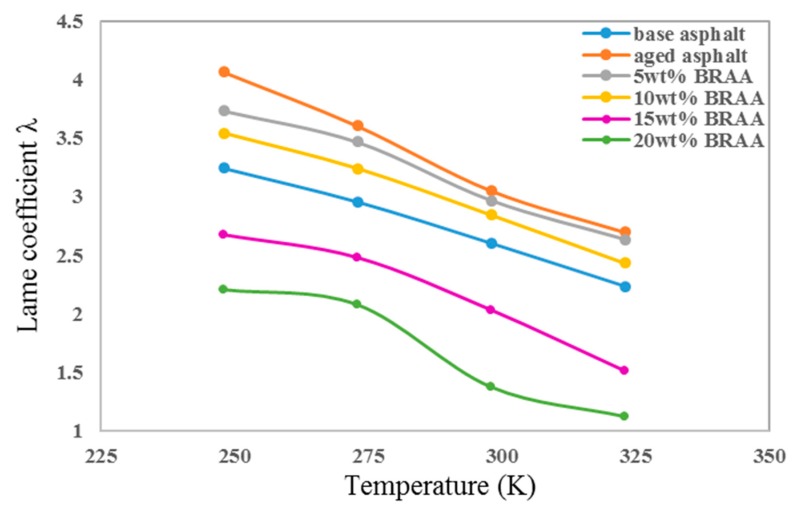
Lame coefficients of asphalt systems.

As shown in Figure 5, the lame coefficient *λ* of asphalt decreased with the increasing temperature. It is demonstrated that temperature could have an influence on the mechanical properties and change the asphalt internal structure. The lame coefficient *λ* of aged asphalt was the highest, and the *λ* of BRAA reduced with the increasing bio-oil content, exhibiting that aging could improve the stiffness of asphalt. But bio-oil would also weaken the resistance to elastic deformation. The lame coefficient *λ* of base asphalt, aged asphalt, 5 wt% BRAA, 10 wt% BRAA, and 15 wt% BRAA at 298 K were 2.60, 3.04, 2.96, 2.84, and 2.02, respectively. The lame coefficient *λ* of 10 wt% BRAA could decrease by 6.6%, while the lame coefficient *λ* of 15 wt% BRAA could decrease by 33.6%. The results indicated that the effect of bio-oil on the lame coefficient λ of asphalt was not linearly taking place. Over 15 wt% bio-oil would significantly reduce the resistance of asphalt to elastic deformation.

**Figure 6:**
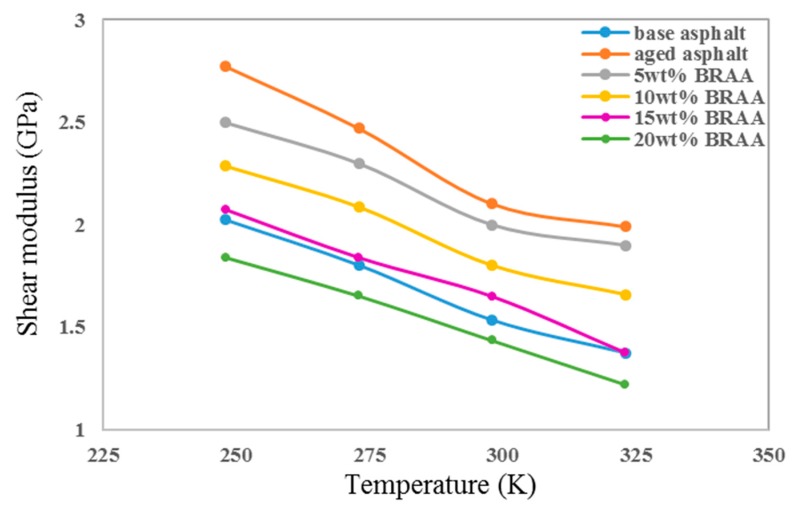
Shear moduli of asphalt systems at different temperatures.

As can be seen in Figure 6, the shear moduli (G) increased after asphalt aging, while the G decreased with the increasing bio-oil addition amount. It indicated that the aging and bio-oil would have influence on the shear moduli. In addition, the G decreased with the increasing temperature, and the bio-oil also decreased the shear moduli of asphalt binder. As can be seen, shear moduli reduced in the order of aged asphalt, 5 wt% BRAA, 10 wt% BRAA, and base asphalt. It indicated that the flexibility of the asphalt decreased after aging, and the bio-oil could recover this change to some extent. The shear moduli of BRAA were close to those of base asphalt when the content of bio-oil reached 15 wt%. The G of 20 wt% BRAA was even lower than that of base asphalt.

**Figure 7:**
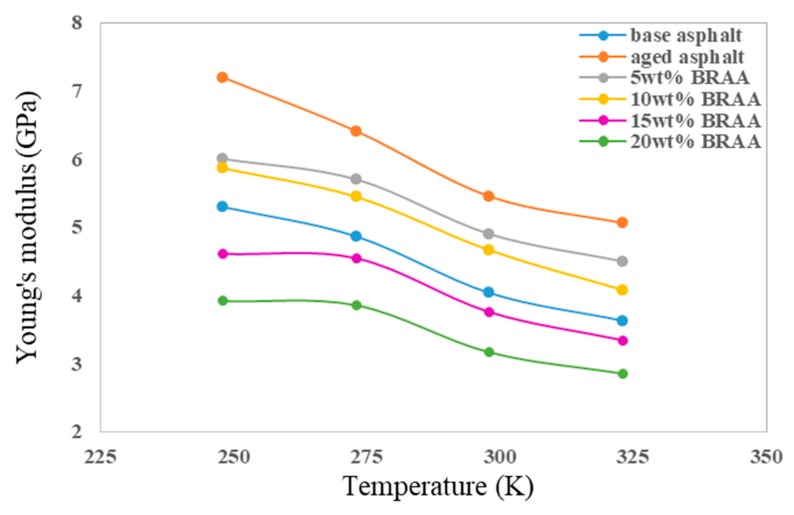
Young’s moduli of asphalt systems at different temperatures.

As shown in Figure 7, the Young’s moduli (K) were calculated according to *λ* and G at different temperatures. The Young’s moduli of aged asphalt were highest, and decreased with the addition of bio-oil, while the K of 5 wt% BRAA and 10 wt% BRAA were bigger than that of base asphalt. The results showed that Young’s modulus of asphalt increased by 35.2% after aging at 298 K, and adding 5 wt% bio-oil could decrease the Young’s modulus of aged asphalt by 10.0%. Bio-oil of 15 wt% could restore the K of aged asphalt restore to its original level.

**Figure 8:**
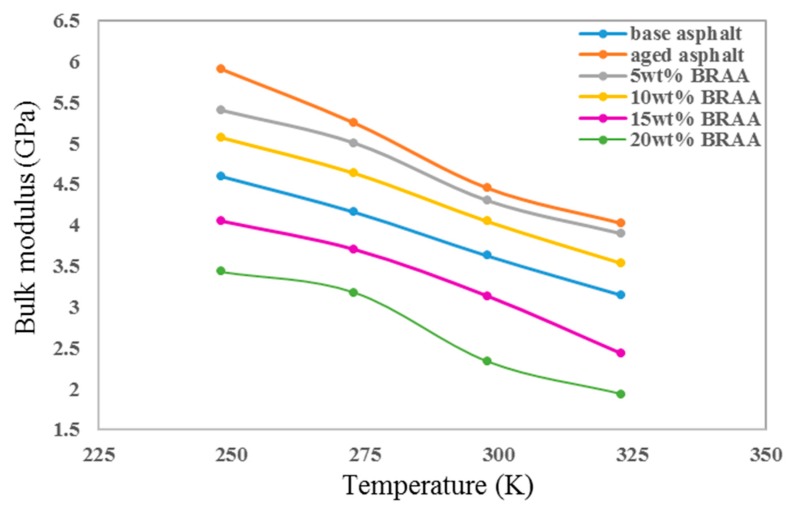
Bulk moduli of asphalt systems at different temperatures.

As shown in Figure 8, the bulk moduli (E) at different temperatures were calculated from *λ* and G. The bulk moduli of asphalt increased after aging. Moreover, the bulk moduli decreased with the additional amount of bio-oil. It indicated that aging and bio-oil could affect the bulk moduli of asphalt. The E of base asphalt increased by 26.2% after aging, and 5 wt% bio-oil could decrease the E of aged asphalt by 4.8%. In addition, when the amount of bio-oil was more than 15 wt%, the BRAA had lower bulk moduli than base asphalt.

**Figure 9:**
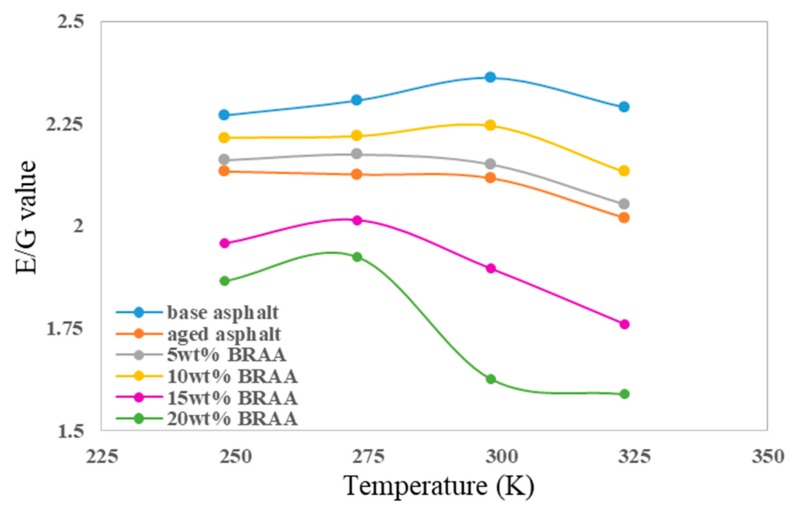
The E/G value of asphalt systems.

The ratio of bulk modulus to shear modulus (E/G) was used to estimate the brittle or ductile behavior of the material. High E/G values represent ductility, while low E/G values represent brittleness. The critical value for separating tough and brittle materials is about 1.75. As shown in Figure 9, calculated E/G value indicated that the base asphalt was ductile. The E/G value of asphalt decreased after aging. The addition of 5 wt% or 10 wt% of bio-oil could recover the ductility of aged asphalt to some extent. The E/G values of base asphalt, aged asphalt, 5 wt% BRAA, and 10 wt% BRAA were 2.361, 2.116, 2.159, and 2.244, respectively. The results showed that the ductility of asphalt decreased by 10.4% after aging, and 10 wt% bio-oil could improve the ductility of aged asphalt by 6.0%. However, 15 wt% bio-oil would further decrease the ductility of aged asphalt. On the other hand, base asphalt had the highest E/G value at 298 K. With the addition of bio-oil, the highest E/G value temperature reduced, which indicated that bio-oil would decrease the temperature stability of asphalt.

## 4. Conclusions

Molecular dynamic simulations were performed in order to investigate the thermal-mechanical properties of bio-oil regenerated aged asphalt (BRAA). Some conclusions were drawn:

(1) The aging process had a significant impact on the thermal properties of asphalt. Aged asphalt had lower specific heat capacity and thermal expansion coefficient than base asphalt. The addition of 5 wt% bio-oil could make the thermal properties of aged asphalt restore to the original level. When the amount of bio-oil was over 10 wt%, the thermal stability of asphalt would be affected, which made the performance of asphalt decrease rapidly with temperature rising.
(2) The aged asphalt had higher elastic constants, Young’s modulus, and bulk modulus than base asphalt, which meant asphalt hardened after aging. The addition of bio-oil would soften the aged asphalt, which reduced the possibility of asphalt cracking. 5 wt% of bio-oil could increase the ductility of aged asphalt by 6.0%. As the amount of bio-oil increased, the structure of asphalt would be affected. When the addition of bio-oil was over 15 wt%, the ductility of aged asphalt would decrease instead.

It should be noticed that the model constructed in this study chose only one kind of molecule to represent each component of asphalt. The results of the simulation could only reflect the actual law to some extent, because asphalt is a complex mixture. The three molecules used to represent bio-oil are the three most abundant in bio-oil. Whether the other ingredients have an influence on the regeneration performance of bio-oil is not certain. Approaching the actual simulation, a more complex system should be built and more components need to be considered.

## 5. Further Research Work

The thermal-mechanical properties of bio-oil regenerated aged asphalt (BRAA) were evaluated, but the data were only based on computer simulations. More work should be done to further verify the rejuvenating effect of bio-oil. The specific heat capacity can be measured by differential scanning calorimetry (DSC), and the thermal expansion coefficient can be tested by Michelson interferometry or quartz dilatometry. Dynamic shear rheological test (DSR) and Dynamic thermomechanical analysis (DMA) can be carried out to measure the shear modulus (G) and bulk modulus (E) of BRAA. A ductility test can be done to verify the relationship between ductility and E/G value. The testing results will be compared to the simulation results. The results of the two may be different in value but have the same trend. Introducing coefficients and increasing the number of samples can improve the accuracy of the simulation. When the accuracy of the model is verified multiple times, the simulation will become the guiding basis for material design.

## Figures and Tables

**Table 1:** Components of asphalt systems.

	Saturates	Aromatics	Resins	Asphaltenes	Acetic Acid	1-Carboxy-2-Propanone	Methanol
base asphalt	13.20%	51.70%	26.90%	8.20%			
aged asphalt	11.60%	37.20%	28.00%	23.20%			
5 wt% BRAA	11.05%	35.43%	26.67%	22.10%	2.86%	1.20%	0.70%
10 wt% BRAA	10.55%	33.82%	25.45%	21.09%	5.47%	2.28%	1.34%
15 wt% BRAA	10.09%	32.35%	24.35%	20.17%	7.84%	3.28%	1.92%
20 wt% BRAA	9.67%	31.00%	23.33%	19.33%	10.02%	4.19%	2.46%
